# Rationale and design of the POLEM trial: avelumab plus fluoropyrimidine-based chemotherapy as adjuvant treatment for stage III mismatch repair deficient or POLE exonuclease domain mutant colon cancer: a phase III randomised study

**DOI:** 10.1136/esmoopen-2019-000638

**Published:** 2020-02-19

**Authors:** David Lau, Eleftheria Kalaitzaki, David N Church, Hardev Pandha, Ian Tomlinson, Nicola Annels, Marco Gerlinger, Francesco Sclafani, Gillian Smith, Ruwaida Begum, Richard Crux, Angela Gillbanks, Sarah Wordsworth, Ian Chau, Naureen Starling, David Cunningham, Tony Dhillon

**Affiliations:** 1 Royal Marsden Hospital NHS Foundation Trust, London, UK; 2 Wellcome Centre for Human Genetics, University of Oxford, Oxford, Oxfordshire, UK; 3 University of Surrey, Guildford, Surrey, UK; 4 Institute of Cancer and Genomic Sciences, University of Birmingham, Birmingham, UK; 5 Institute of Cancer Research, London, UK; 6 Nuffield Department of Population Health, University of Oxford, Oxford, Oxfordshire, UK; 7 Royal Surrey NHS Foundation Trust, Guildford, UK; 8 University of Surrey, Guildford, United Kingdom

**Keywords:** colon cancer, mismatch repair, adjuvant therapy, POLE mutation, microsatellite instability

## Abstract

**Background:**

10%–15% of early-stage colon cancers harbour either deficient mismatch repair (dMMR), microsatellite instability high (MSI-H) or *POLE* exonuclease domain mutations, and are characterised by high tumour mutational burden and increased lymphocytic infiltrate. Metastatic dMMR colon cancers are highly sensitive to immune checkpoint inhibition, and recent data show *POLE*-mutant tumours are similarly responsive. We are conducting a phase III randomised trial to determine if the addition of the anti-PD-L1 antibody avelumab following adjuvant chemotherapy improves disease-free survival (DFS) in patients with stage III dMMR/MSI-H or *POLE* mutant colon cancer and is a cost-effective approach for the UK National Health Service (NHS).

**Methods:**

We are recruiting patients with completely resected, stage III colon cancer confirmed to have dMMR/MSI-H, locally or *POLE* exonuclease domain mutation on central testing. Eligible patients are randomised in a 1:1 ratio to standard fluoropyrimidine-based chemotherapy (capecitabine, oxaliplatin for 12 weeks or capecitabine for 24 weeks) or chemotherapy, followed by avelumab (10 mg/kg, 2 weekly for 24 weeks). Stratification is by chemotherapy received and MMR/MSI-H status. The primary endpoint is DFS. Secondary endpoints include overall survival, toxicity, quality of life and health resource use. The 3-year DFS rate in the control arm is expected to be ~75%. Avelumab is expected to improve the 3-year DFS rate by 12% (ie, 87%). Target accrual is 402 patients, which provides 80% power to detect an HR of 0.48 for DFS at a two-sided alpha of 0.05. This national, multicentre phase III trial is sponsored by the Royal Marsden NHS Foundation Trust and it is anticipated that approximately 40 centres in the UK will participate. This study opened to recruitment in August 2018.

**Trial registration number:**

NCT03827044

**Key questions**

**What is already known about this subject?**

- Fluoropyrimidine-based adjuvant chemotherapy following surgery is the current standard of care for stage III colon cancer.
- Immune checkpoint therapy is effective in the treatment of deficient mismatch repair (dMMR)/microsatellite instability high (MSI-H) colorectal cancer (CRC) in the advanced setting. *POLE* mutant CRC (mCRC) has also been proposed as a type of mCRC, which is also responsive to immunotherapy.
- To our knowledge, there is no mature randomised clinical data to support the use of immune checkpoint inhibitors in the curative setting such as dMMR/MSI-H or *POLE* mutant stage III colon.

**What does this study add?**

- The POLEM trial is an open-label, multicentre, randomised, phase III study testing the efficacy of the immune checkpoint inhibitor avelumab (anti-PD-L1) following standard adjuvant chemotherapy in dMMR/MSI-H or *POLE* mutant stage III colon cancer.
- Eligible patients are randomly allocated to receive investigator choice chemotherapy (12 weeks of capecitabine, oxaliplatin or 24 weeks capecitabine), followed by avelumab for 24 weeks or chemotherapy alone.
- The recruitment aim is 402 patients and the study is currently open in the UK with potential for international collaboration.

**Key questions**

**How might this impact on clinical practice?**

- The results from this study will determine whether immune checkpoint therapy such as avelumab (anti-PD-L1) should be added to standard adjuvant chemotherapy in deficient mismatch repair/microsatellite instability high or POLE mutant stage III colon cancer.

## Introduction

Colorectal cancer (CRC) is the third most common cancer, with a worldwide annual incidence of over 1.2 million cases and a mortality rate of approximately 50%.^1 2^ Around, 80% of patients with CRC have localised and resectable disease at diagnosis, with 5-year survival varying from 90% in stage I to 70%–80% in stage II and 40%–65% in stage III disease. The risk of recurrence also depends on the pathological stage of the primary tumour (30% in stage II and 50% in stage III) and is higher within the first 2 years after surgery.^3^ The treatment of resectable disease is surgery ±adjuvant fluoropyrimidine-based chemotherapy depending on the pathological stage. To improve these survival statistics, there is a need for new treatments and predictive and prognostic biomarkers that can identify patients who are most likely to benefit.

The DNA mismatch repair (MMR) machinery is essential for maintenance of genomic integrity. Defects in DNA MMR can occur either at the germline (Lynch syndrome) or epigenetic level.^4^ Deficiency MMR (dMMR) results in a failure to repair DNA replication errors, manifest as an increased frequency of somatic mutations^5^—typically 10 to 100-foldgreater than MMR proficient/microsatellite stable (pMMR/MSS) CRC.^6–8^ dMMR/microsatellite instability high (MSI-H) is more common among stage II (20%) than stage III (12%) and less frequent among stage IV CRC (4%).^9 10^ dMMR/MSI-H CRCs tend to be right sided, high grade and have mucinous phenotypes and prominent numbers of tumour-infiltrating lymphocytes.^11^ The mean disease-free survival (DFS) of stage III dMMR/MSI-H CRC is around 73% and 5-year overall survival (OS) is 83%.^12^

The management of metastatic dMMR/MSI-H CRC has recently been transformed by clinical data demonstrating remarkable clinical benefit of PD-1 inhibitors in this setting.^13–16^ Mechanistically, this is thought to relate to the high number of neoantigens in these tumours,^13^ and the reversal of the strong upregulation of immune checkpoints (eg, PD-1, PD-L1, cytotoxic T lymphocytes-associated protein-4 (CTLA-4), LAG-3 and IDO) which may overcome a phenomenon referred to as ‘adaptive immune resistance’. These data, together with evidence that increased PD-L1 expression is associated with poor prognosis in stage III CRC,^15^ provide a compelling rationale for testing the efficacy of immune checkpoint inhibition in stage III dMMR/MSI-H CRC.

Importantly, National Institute for Health and Care Excellence (NICE, UK) guidance from 2017 recommends MMR or microsatellite instability testing should be undertaken for all patients with CRC, when first diagnosed, using immunohistochemistry or PCR for Lynch syndrome.^17^

The proofreading exonuclease activity intrinsic to the replicative DNA polymerases *POLE* and *POLD1* is critical in the maintenance of DNA replication fidelity and prevention of mutagenesis. The relevance of this to human cancer was confirmed by the demonstration that germline mutations in the *POLE* and *POLD1* exonuclease domains cause intestinal polyposis and early-onset cancer,^18^ and the demonstration of somatic *POLE* exonuclease domain mutations in CRC by The Cancer Genome Atlas (TCGA).^8^ In the TCGA study, *POLE*-mutant cancers were found to have and exceptionally high number of somatic mutations (over 50 per 10^6^ bases), in the absence of MMR defects.^18 19^ Further analysis of these cases revealed higher expression of immune checkpoints, including PD-L1 and PD-L2, than either dMMR/MSI-H or pMMR/MSS cancers.^20^ Similarly, *POLE* mutant cancers also showed higher expression of T cell markers such as CD8A, PD-1 and CTLA-4 suggesting the presence of a pre-existing T cell infiltrate.^20^

TCGA data suggest that *POLE* exonuclease domain mutant somatic mutations are in the range of 1%–3% of patients,^8^ however, published and unpublished data^21^ show that in the under 50 years the incidence of *POLE* mutations is 8%–10%. While somatic *POLE* mutation portends reduced risk of relapse in stage II CRC, the association with favourable prognosis appears to be lost in stage III disease.^20 22^ These tumours with a so-called ‘ultramutated’ phenotype may be very responsive to checkpoint inhibition for similar reasons to those thought to underlie the increased sensitivity of dMMR cases.^18^ Indeed, a recent case report suggests this is the case.^23^

The aim of this study is to determine whether dMMR/MSI-H and/or *POLE* exonuclease domain mutant stage III colon cancer patients gain clinical benefit (ie, improvement in disease-free and OS) from PD-L1 inhibition after standard fluoropyrimidine-based adjuvant chemotherapy and to examine whether this is a cost-effective approach for the National Health Service.

Avelumab is a humanised monoclonal antibody, which binds PD-L1 and blocks the interaction between PD-L1 and PD-1. This removes the suppressive effects of PD-L1 on anti-tumour CD8 +T cells, resulting in the restoration of cytotoxic T cell response.

The rationale of giving avelumab after standard adjuvant chemotherapy to this well-defined, molecularly selected, group is based on the observation that dMMR/MSI-H and *POLE* exonuclease domain mutant CRCs have a highly and ultramutated genetic profile, respectively, thus leading to a high number of neoantigens with associated over expression of immune checkpoint-related proteins. This profile is expected to be highly responsive to checkpoint inhibition as suggested by data of PD-1 inhibitors in dMMR/MSI-H metastatic CRC.^14–16^

If this study meets the primary endpoint, using avelumab in the adjuvant setting following standard chemotherapy would become the standard of care for patients with dMMR/MSI-H and/or *POLE* exonuclease domain mutant colon cancers. Furthermore, given the availability of molecular markers for patient selection, funders of healthcare would be more likely to fund this treatment.

This study also provides a unique opportunity to conduct translational research analyses on pretreatment and post-treatment tumour tissue samples and blood samples from dMMR/MSI-H or *POLE* mutant CRC patients treated with the immune checkpoint inhibitor avelumab.

## Methods

### Study design and treatment

POLEM is an open-label, multicentre, randomised, phase III trial comparing standard fluoropyrimidine-based adjuvant chemotherapy, followed by avelumab (experimental arm) with standard fluoropyrimidine-based adjuvant chemotherapy alone (control arm) in patients who have undergone radical surgical resection for stage III dMMR/MSI-H or *POLE* exonuclease domain mutant colon cancer. Eligible patients (table 1) will be stratified in a 1:1 ratio for dMMR/MSI-H status and type of adjuvant chemotherapy (ie, 24 weeks of single agent capecitabine chemotherapy vs 12 weeks of capecitabine, oxaliplatin chemotherapy).

**Table 1:** POLEM trial inclusion and exclusion criteria

Inclusion criteria	Exclusion criteria
Male or female subjects aged ≥18 years ECOG PS 0/1 Histologically proven, stage III (ie, any T, N1 or N2, M0) adenocarcinoma of the colon (as defined by the presence of the inferior pole of the tumour above the peritoneal reflection, ie, at least 15 cm from the anal margin). Fully surgically resected tumour with clear resection margins (ie, >1 mm). Locally confirmed defective mismatch repair (MMR) tumour (as defined by the lack of staining on either preoperative biopsy samples or resection specimens of at least one of the following proteins: MLH1, MSH2, MSH6, PMS2), locally confirmed microsatellite instability high, or centrally confirmed POLE exonuclease domain mutated tumour (in subjects <50 years old with locally confirmed proficient MMR tumours). Absence of metastases as shown by postoperative CT and/or MRI scan. Absence of major postoperative complications or other clinical conditions that, in the opinion of the investigator, would contraindicate adjuvant chemotherapy. Adequate haematological function defined by absolute neutrophil count ≥1.5×10^9^/L, platelet count ≥100×10^9^/L and haemoglobin ≥9 g/L (blood transfusion before recruitment is allowed). Adequate hepatic function defined by a total bilirubin level ≤1.5× the upper limit of normal (ULN) range and AST and ALT levels ≤2.5 × ULN. Adequate renal function defined by an estimated creatinine clearance ≥30 mL/min according to the Cockcroft-Gault formula (or local institutional standard method). Negative serum or urine pregnancy test at screening for women of childbearing potential. Fertile men and women must agree to take highly effective contraceptive precautions during, and for 6 months after the last dose of chemotherapy or for 30 days after the last dose of avelumab.	Rectal tumours (as defined by the presence of the inferior pole of the tumour below the peritoneal reflection, ie,<15 cm from the anal margin). Inability to start adjuvant chemotherapy within 12 weeks after surgery. Administration of neoadjuvant systemic chemotherapy or radiotherapy before surgical resection of colon cancer. Prior organ transplantation, including allogeneic stem-cell transplantation. Significant acute or chronic infections including, among others: Positive test for HIV or known AIDS. Positive test for Hepatitis B (HBV) surface antigen or anti-HCV antibody and confirmatory Hepatitis C (HCV) RNA test. Active autoimmune disease that might deteriorate when receiving an immunostimulatory agent: Subjects with diabetes type I, vitiligo, psoriasis, hypothyroid or hyperthyroid disease not requiring immunosuppressive treatment are eligible. Subjects requiring hormone replacement with corticosteroids are eligible if the steroids are administered only for the purpose of hormonal replacement and at doses ≤10 mg/day of prednisone or equivalent. Administration of steroids through a route known to result in a minimal systemic exposure (topical, intranasal, intro-ocular or inhalation) are acceptable. Known severe hypersensitivity reactions to monoclonal antibodies (grade ≥3 NCI-CTCAE V.4.0), any history of anaphylaxis, or uncontrolled asthma (ie, 3 or more features of partially controlled asthma). Persisting toxicity related to prior therapy of grade >1 NCI-CTCAE V.4.0; however, alopecia and sensory neuropathy grade ≤2 is acceptable unless oxaliplatin administration is planned as part of the adjuvant treatment. Pregnancy or lactation. Known alcohol or drug abuse. Clinically significant (ie, active) cardiovascular disease: cerebral vascular accident/stroke (<6 months prior to enrolment), myocardial infarction (<6 months prior to enrolment), unstable angina, congestive heart failure (≥New York Heart Association Classification Class II) or serious cardiac arrhythmia requiring medication. Prior myocarditis. Known history of immune colitis, immune pneumonitis, pulmonary fibrosis or other medical conditions (eg, inflammatory bowel disease, uncontrolled asthma), which, in the opinion of the investigator, might impair the subject’s tolerance of trial treatment. Any psychiatric condition that would prohibit the understanding or rendering of informed consent. Vaccination within 4 weeks of the first dose of avelumab and while on trial is prohibited except for administration of inactivated vaccines. Other invasive malignancy within 2 years except for non-invasive malignancies such as cervical carcinoma in situ, non-melanomatous carcinoma of the skin or ductal carcinoma in situ of the breast that has/have been surgically cured.

ALT, alanine transaminase; AST, aspartate transaminase; ECOG PS, Eastern Cooperative Oncology Group performance status; NCI-CTCAE, National Cancer Institute Common Terminology Criteria for Adverse Events.

According to the statistical design, 402 patients (201 per arm) are to be randomised. It is expected that approximately 4000 participants will need to be screened in order to recruit 402 patients to the study, assuming an incidence of dMMR/MSI-H of 10%–15% and an incidence of *POLE* mutations of 8%–10% in patients under 50 years of age.^21^ Considering the time required to obtain local approval and to initiate all participating centres, the study is expected to take up to 36 months to complete accrual.

There are no proscriptive criteria for surgical resection of the primary tumour in this trial. However, it is expected that resection of the tumour will be undertaken in the elective setting by a colorectal specialist surgeon.

Tumour MMR/MSI status will be routinely tested locally as per NICE guidelines^17^ either in the preoperative biopsy or resection specimen. Patients whose tumours are dMMR/MSI-H can provide consent to the main study and undergo the study screening procedures. If they are found to fulfil all eligibility criteria, then they will be randomised. Patients who are aged below 50 years and whose tumours are pMMR/MSS, will be asked to sign a pre-screening consent for the centralised analysis of *POLE* exonuclease domain mutations. Those who have tumours harbouring these mutations can sign the main study consent and undergo the study screening procedures. If they are found to fulfil all eligibility criteria, then they will be randomised.

All eligible patients who are randomised will receive standard fluoropyrimidine-based adjuvant chemotherapy for 12 or 24 weeks depending on the decision of the local investigator. The choice of adjuvant chemotherapy (ie, 24 weeks of single agent fluoropyrimidine chemotherapy or 12 weeks of doublet, oxaliplatin-based chemotherapy) must be declared by the investigator at study entry before randomisation. The adjuvant chemotherapy regimen and MMR/MSI status will be used as stratification factors.

**Figure 1:**
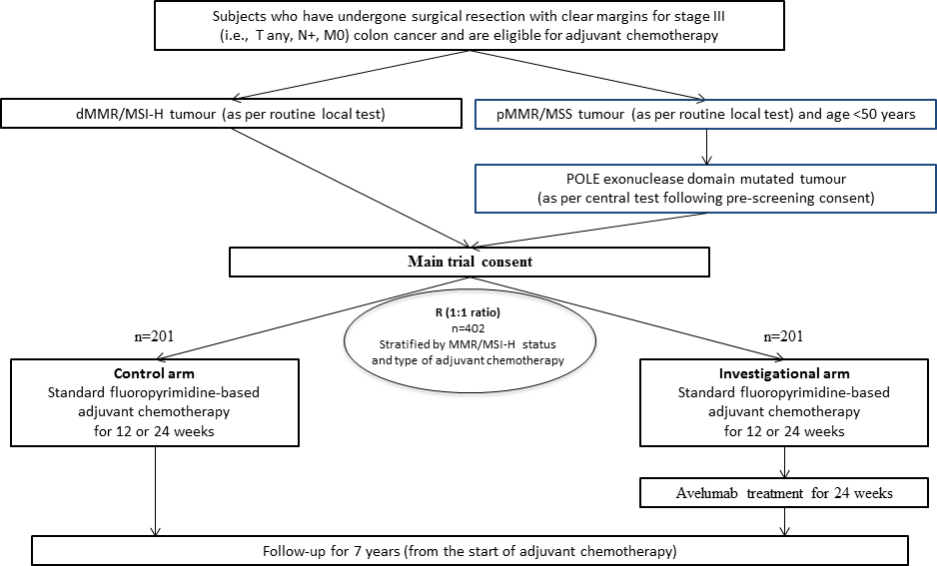
Study design of the POLEM trial. *Capecitabine chemotherapy for 24 weeks or capecitabine/oxaliplatin for 12 weeks. dMMR, deficient mismatch repair; MSI-H, microsatellite instability high; MSS, microsatellite stable; pMMR, proficient mismatch repair.

At the end of adjuvant chemotherapy, patients who are randomised to the investigational arm will receive additional 24 weeks of treatment with avelumab (figure 1).

### Study objectives and endpoints

#### Primary endpoint

The primary endpoint is DFS at 3 years. This is defined as the proportion of subjects who are alive and disease free at 3 years. For subjects who neither progressed nor died, their DFS time will be censored at the last date of contact. The main analysis will take place once 60 events have occurred.

Relapse will be based on investigator reported imaging assessments or colonoscopy. Where a patient has neither progressed nor died, their DFS time will be censored at the last date of contact (known to be alive and relapse free).

#### Secondary endpoints

- Median DFS will be reported with a 95% CIs in each group.
- OS at 5 and 7 years is defined as the proportion of patients who are alive at 5 and 7 years, respectively. Patients alive at the time of analysis will be censored at the date of last follow-up. Analysis methods for OS will follow those of the primary endpoint DFS (see primary endpoints above).
- Median OS will also be reported as DFS section above.
- Adverse events as assessed by National Cancer Institute Common Terminology Criteria for Adverse Events V.4.0
- Health-related quality of life based on EORTC QLQ C-30 and 5-level EuroQol 5 Dimensions: QLQ C-30 will be summarised over time, as per the standard scoring manual, for each of the 15 domains using mean and SD. Statistical analysis will consist of linear mixed models and the mean difference between treatments for each domain score will be presented.
- Quality-adjusted life years (QALYs).
- Health resource use: health resource use will be collected prospectively postrandomisation a healthcare services resource questionnaire designed for the trial.

Resource use and associated costs will be combined with quality-of-life data to provide a cost per QALY for fluoropyrimidine-based adjuvant chemotherapy, followed by avelumab compared with standard fluoropyrimidine-based adjuvant chemotherapy alone. In terms of analysis, Kaplan-Meier plots will be used to summarise the survival estimates and report the DFS rate at 3 years along with a 95% CIs in each group. DFS rates between groups will be compared using the log-rank test. In addition, Cox proportional hazards (PH) model (or alternative methods, eg, method of Klein) will be used to compare the DFS rates overall and at 3 years adjusting for prespecified covariates. A test for non-PH will also be carried out.

The primary analysis for DFS outcome will be unadjusted and will be based on the intention-to-treat (ITT) population with a sensitivity analysis in the modified ITT population and in the per-protocol population.

### Sample size calculation

The 3-year DFS rate in the control arm is expected to be about 75%.^24^ The experimental treatment (fluoropyrimidine-based chemotherapy followed by avelumab) is expected to improve the 3-year DFS rate by 12% (to 87%), corresponding to an HR of 0.48. This effect size is justifiable since the population is highly enriched (with dMMR/MSI-H and *POLE* mutant patients) and second, a large effect size will also allow potential for cost-effectiveness of the experimental intervention.

Therefore, a sample size of at least 171 per group (342 in total) is required (60 DFS events in total) to reject the null hypothesis of:

H_0_; HR=1 vs H_1_: HR HR≠1 with at least 80% power assuming a two-sided 5% type I error.

Assuming further a 15% drop-out rate (lost to follow-up rate) a total sample size of 402 will be required (201 per group in a 1:1 randomisation).

### Planned recruitment

The incidence of stage III dMMR/MSI-H CRC is reported to be about 1753 per year in the UK (England and Scotland) (based on 41 112 CRC patients diagnosed in UK in 2013 (latest figures), 23.7% of these are stage III and 18% of them are dMMR/MSI-H). Assume some 20% of these may fail screening/eligibility criteria, the pool of patients available for recruitment is likely to be approximately 1500 per year.

With a target of 402 patients, assuming recruitment over 36 months, on average, about 11 patients per month are anticipated (ie, 400/36=~11/ month). At least 30 sites are planned to be activated. Hence, on average, <1 patient per month is required per site. Assuming that recruitment will not be heavily competitive as the number of CRC trials in this setting are few, the target sample size is expected to be reached.

## Discussion

The POLEM trial is one of two studies evaluating checkpoint inhibitor drugs in the adjuvant setting in stage III dMMR/MSI-H colon cancer; the other being the US-based ATOMIC study (ClinicalTrials.gov Identifier: NCT02912559). POLEM is distinct from ATOMIC in that it also includes *POLE* mutant colon cancers and has been designed to incorporate the recent IDEA collaboration trial data comparing 3 months vs 6 months fluoropyrimidine-based chemotherapy.^25^ The study opened in August 2018 and will be conducted in over at least 30 UK sites with potential for international collaboration. We anticipate that the findings could change the standard of care in this molecularly defined stage III colon cancer population.
